# Improving the efficiency and accuracy of cardiovascular magnetic resonance with artificial intelligence—review of evidence and proposition of a roadmap to clinical translation

**DOI:** 10.1016/j.jocmr.2024.101051

**Published:** 2024-06-22

**Authors:** Qiang Zhang, Anastasia Fotaki, Sona Ghadimi, Yu Wang, Mariya Doneva, Jens Wetzl, Jana G. Delfino, Declan P. O’Regan, Claudia Prieto, Frederick H. Epstein

**Affiliations:** a Oxford Centre for Clinical Magnetic Resonance Research, Division of Cardiovascular Medicine, Radcliffe Department of Medicine, University of Oxford, Oxford, UK; b Big Data Institute, University of Oxford, Oxford, UK; c School of Biomedical Engineering and Imaging Sciences, King’s College London, London, UK; d Royal Brompton Hospital, Guy’s and St Thomas’ NHS Foundation Trust, London, UK; e Department of Biomedical Engineering, University of Virginia, Charlottesville, VA, USA; f Philips Innovative Technologies, Hamburg, Germany; g Siemens Healthineers AG, Erlangen, Germany; h US Food and Drug Administration, Center for Devices and Radiological Health (CDRH), Office of Science and Engineering Laboratories (OSEL), Silver Spring, MD, USA; i MRC Laboratory of Medical Sciences, Imperial College London, London, UK; j School of Engineering, Pontificia Universidad Católica de Chile, Santiago, Chile

**Keywords:** Cardiovascular magnetic resonance, Artificial intelligence, Deep learning, Clinical translation, Review, Roadmap

## Abstract

**Background:**

Cardiovascular magnetic resonance (CMR) is an important imaging modality for the assessment of heart disease; however, limitations of CMR include long exam times and high complexity compared to other cardiac imaging modalities. Recently advancements in artificial intelligence (AI) technology have shown great potential to address many CMR limitations. While the developments are remarkable, translation of AI-based methods into real-world CMR clinical practice remains at a nascent stage and much work lies ahead to realize the full potential of AI for CMR.

**Methods:**

Herein we review recent cutting-edge and representative examples demonstrating how AI can advance CMR in areas such as exam planning, accelerated image reconstruction, post-processing, quality control, classification and diagnosis.

**Results:**

These advances can be applied to speed up and simplify essentially every application including cine, strain, late gadolinium enhancement, parametric mapping, 3D whole heart, flow, perfusion and others. AI is a unique technology based on training models using data. Beyond reviewing the literature, this paper discusses important AI-specific issues in the context of CMR, including (1) properties and characteristics of datasets for training and validation, (2) previously published guidelines for reporting CMR AI research, (3) considerations around clinical deployment, (4) responsibilities of clinicians and the need for multi-disciplinary teams in the development and deployment of AI in CMR, (5) industry considerations, and (6) regulatory perspectives.

**Conclusions:**

Understanding and consideration of all these factors will contribute to the effective and ethical deployment of AI to improve clinical CMR.

## Graphical abstract

**Figure:**
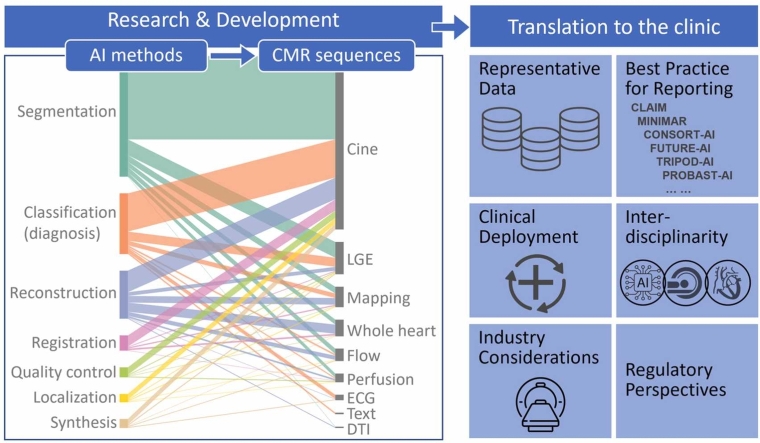

## 1 Background

Cardiovascular magnetic resonance (CMR) is the most comprehensive non-invasive technique for assessing cardiac structure, function, perfusion, tissue characterization, and cardiovascular hemodynamics, providing high-quality data for diagnosing heart disease and predicting outcomes. CMR is widely used in clinical practice, but its efficiency and accessibility are hindered by the complexity of performing CMR studies, long exam times, high cost, and the requirement for manual image analysis by experts. Artificial intelligence (AI), particularly deep learning (DL), has demonstrated remarkable progress recently and holds great potential to overcome many of the limitations of CMR. However, despite the large volume of research studies related to this topic, translation of AI methods into the real-world clinical CMR workflow remains challenging. In this article, we use the terms AI, DL, and machine learning (ML), which have distinct meanings that have been previously defined [1]. Briefly, and in practical terms for this paper, AI is a broad umbrella term that refers to the ability of computers to mimic human intelligence, DL refers to deep neural networks that learn from large datasets, and we use ML to refer to shallow learning algorithms, such as support vector machines and decision trees.

A number of papers have reviewed research in this field. Some focused on AI methods for specific CMR sequences, such as parametric mapping [2], perfusion [3], fingerprinting [4], and late gadolinium enhancement (LGE) [5]. Other review papers summarized the state-of-the-art for specific tasks, such as reconstruction [6], segmentation [7], [8], motion and deformation analysis [9], and outcome prediction [10]. Review papers summarizing AI applications for specific diseases, such as myocardial infarction (MI) [11] and dilated cardiomyopathy [12], have also been written. Additionally, there are publications that provide an overview of AI basics with exemplar CMR applications [13], [14] or in the context of multi-modality imaging [15]. Most of these prior articles focus on reviewing evidence of a specific aspect at the research and development stage, while consideration of a roadmap toward their clinical adoption is in demand, but absent.

**Fig. 1:**
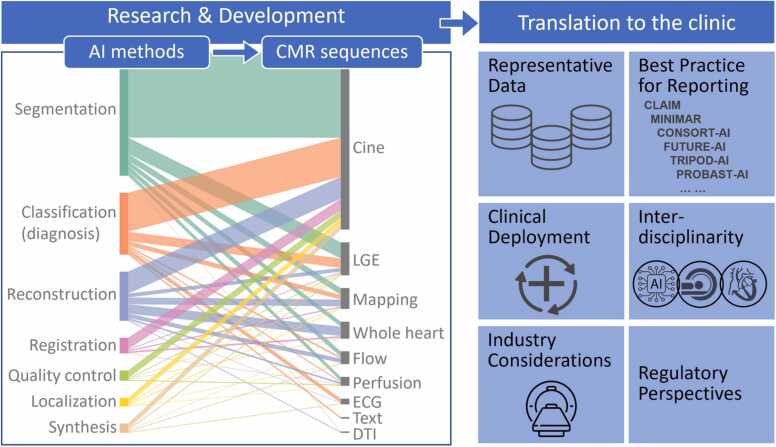
Graphical abstract. This paper introduced image processing tasks that can be accelerated or automated with AI and reviewed the applications of AI in common CMR sequences. It then discussed important AI-specific issues when translating AI CMR methods to clinical practice. *AI* artificial intelligence, *CMR* cardiovascular magnetic resonance, *DTI* diffusion tensor imaging, *ECG* electrocardiogram, *LGE*: late gadolinium enhancement. For abbreviations of checklist, see section 3.2 below.

The intention of this article is not to be overly technical but to provide an overarching introduction of cutting-edge and illustrative examples for the reader hoping to understand the general concepts and clinical applications in this rapidly growing area. Specifically, we introduce image reconstruction, post-processing, quality control (QC), classification, and prognostication tasks that can be accelerated, improved, and/or automated with AI. We then review the roles and applications of AI in common CMR sequences. Beyond reviewing the literature, we brought together CMR clinicians, AI scientists, CMR physicists, industry partners, and experts in regulatory sciences to envision a roadmap to clinical translation of AI CMR methods. The authors convened a meeting of the Society for Cardiovascular Magnetic Resonance (SCMR) AI Special Interest Group during the 2023 SCMR annual scientific sessions (San Diego, LA, 2023) where more than 50 people gathered and provided input. The authors hope that this article can summarize recent developments in AI applied to CMR and suggest approaches to accelerate the adoption of AI in clinical CMR to gain the advantages offered by AI and to do so in a manner that is fair, responsible, and equitable. A graphical abstract is provided in Fig. 1.

## 2 Review of literature—current AI CMR methods and applications

**Fig. 2:**
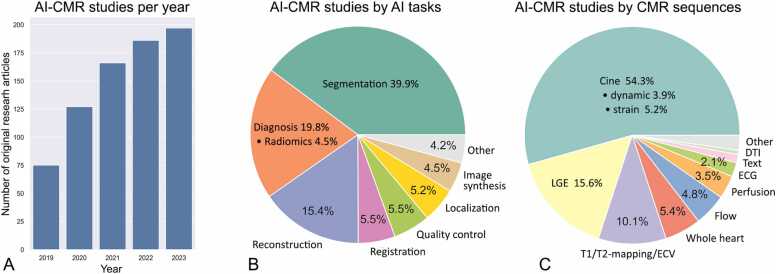
Five-year literature review between 2019 and 2023. (A) Number of original AI CMR research papers per year (totaling N=751 papers). (B) Published AI CMR studies categorized by AI tasks. (C) Published AI CMR studies categorized by CMR sequences. “Whole-heart” imaging includes 3D coronary MR angiography. “Flow” includes 2D phase-contrast imaging and 4D flow. “Text” includes patient characteristics, history, and CMR reports. *2D*: two-dimensional, *3D*: three-dimensional, *4D*: four-dimensional; *AI*: artificial intelligence, *CMR*: cardiovascular magnetic resonance, *DTI*: diffusion tensor imaging, *ECG*: electrocardiogram, *ECV*: extracellular volume, *LGE*: late gadolinium enhancement.

**Fig. 3:**
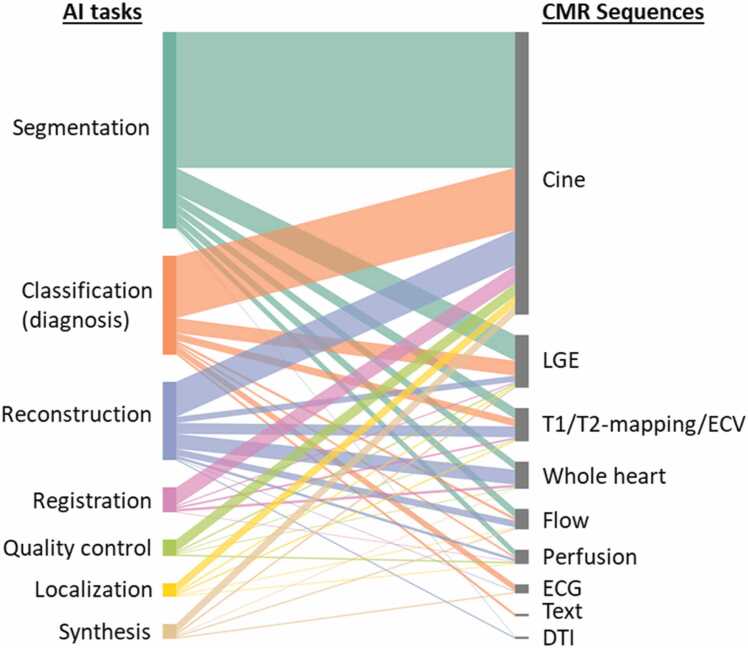
Visualization of 751 original studies applying AI to CMR between 2019 and 2023. Segmentation of cine imaging is the most intensively studied as a crucial step for assessing cardiac structure and function. Additionally, AI has been applied to almost all common CMR sequences to automate the segmentation, classify diseases, accelerate reconstruction, perform image registration, improve image quality, localize anatomies, and synthesize new data. *AI* artificial intelligence, *CMR* cardiovascular magnetic resonance, *DTI* diffusion tensor imaging, *ECG* electrocardiogram, *ECV* extracellular volume, *LGE* late gadolinium enhancement.

To conduct this narrative review, we performed comprehensive PubMed searches using varying combinations of keywords [“artificial intelligence”, “machine learning”, “deep learning”] and keywords [“CMR”, “cardiac MRI”, “cardiovascular MRI”] within the 5-year period of 2019 and 2023. Articles studying primarily other modalities or organs were manually excluded, and the search was supplemented by manually identified papers, leading to an aggregate of 751 original research articles that were examined and contributed to our synopsis. The number of AI CMR publications per year is increasing rapidly, reflecting the growing interest and activity in this field (Fig. 2A). All research articles were dissected to show the spectrum of these studies in terms of AI tasks (Fig. 2B) and CMR sequences (Fig. 2C). The AI CMR literature (N = 751 articles) was further visualized in a bipartite graph, which unveiled a comprehensive translation of AI approaches and tasks to various CMR sequences (Fig. 3). Based on this framework, our review of the literature is presented in the following sub-sections.

### 2.1 AI tasks for CMR

AI can significantly impact the entire CMR workflow, including image reconstruction, image analysis, QC, and diagnosis/prognosis. Recent research advancements promise to speed up scan protocols, automate CMR planning and image processing, improve image quality, and support medical diagnosis and prognostication.

#### 2.1.1 Acceleration and reconstruction

**Fig. 4:**
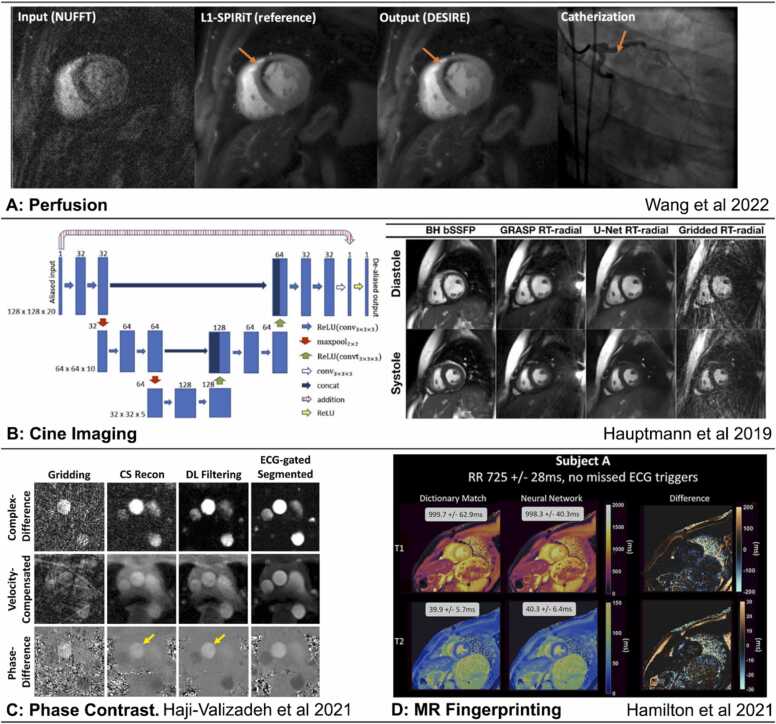
AI in CMR image reconstruction. (A) DEep learning-based rapid Spiral Image REconstruction (DESIRE) technique for high-resolution spiral first-pass myocardial perfusion imaging with whole-heart coverage, reprinted with permission [32]. (B) Convolutional neural network for reconstruction of real-time radial data acquired in 18 ± 3 s [33]. (C) Deep-learning framework for removal of aliasing artifacts from accelerated radial real-time phase-contrast MRI, reprinted with permission [34]. (D) CMR fingerprinting T1 and T2 maps correspond to dictionary-based pattern matching reconstruction (>4 min per slice) and the time-efficient deep-learning based reconstruction (336 ms per slice), reprinted with permission [35]. *AI:* artificial intelligence, *CMR:* cardiovascular magnetic resonance, *DL:* deep learning, *ECG:* electrocardiogram, *MRI:* magnetic resonance imaging, *NUFFT:* non-uniform fast Fourier transform*, SPIRiT:* self-consistent parrallel imaging, *RT:* real time, *CS:* compressed sensing, *L!-SPIRIT:* L1 Iterative self-consistent parallel imaging reconstruction, *ReLU:* Rectified Linear Unit, BH bSSFP*:* breathhold balanced steady state free precession, *GRASP RT:* Golden-angle radial sparse parallel real-time, *U-Net RT:* U-Net real-time.

Due to the sequential acquisition of data samples in k-space and electrocardiogram (ECG) synchronization, CMR image acquisition is inherently slow. One approach to accelerate the acquisition is to undersample k-space. However, undersampling introduces aliasing artifacts when the image is reconstructed. A variety of data acquisition and image reconstruction techniques have been developed to produce images of acceptable quality from undersampled data. These techniques exploit coil sensitivity profiles (parallel imaging) [16], sparsity of data in a transform domain (compressed-sensing) [17], [18], [19], [20], and low-rank properties in spatial and/or temporal dimensions [21]. Those approaches, however, come at the cost of high computational burden and long reconstruction times, and are dependent on the choice of the reconstruction parameters, which might not perfectly model the spatiotemporal complexity of CMR imaging [22], [23], [24]. DL approaches have recently been proposed to learn up-front the non-linear optimization processes employed in CMR reconstruction, making use of large datasets to learn the key reconstruction parameters and priors. These methods differ in terms of their intended tasks and include image denoising using image-to-image regressions [25]; direct mapping from acquired k-space to the reconstructed image [26], [27]; physics-based k-space learning or unrolled optimizations [28], [29], [30]; and combinations of these. An alternative approach for accelerating image acquisition is the use of DL–based super-resolution, where images are acquired at a low resolution, with or without undersampling, and retrospectively reconstructed to the high-resolution target [31]. Examples demonstrating various CMR sequences are illustrated in Fig. 4.

#### 2.1.2 Segmentation

**Fig. 5:**
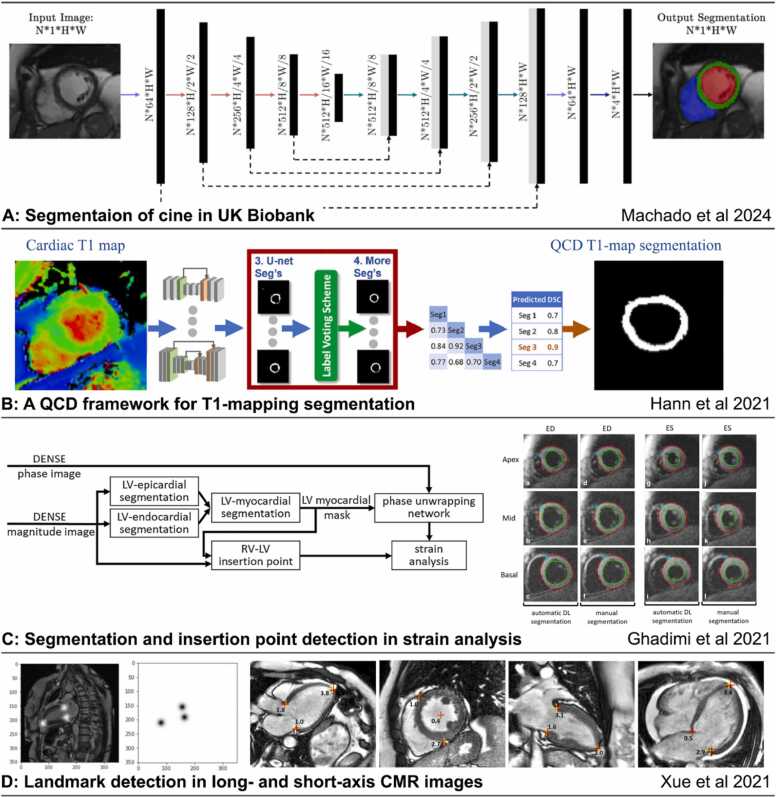
AI in CMR image segmentation and landmark localization. (A) A recent convolutional network was developed to segment cine images from the UK Biobank, reprinted with permission [55]. (B) A quality-control driven framework for segmentation of T1-mapping [38]. (C) A deep-learning approach for landmark localization and LV segmentation for automated strain analysis of DENSE CMR [36]. (D) Landmark detection in long- and short-axis CMR images [56]. *AI:* artificial intelligence, *CMR:* cardiovascular magnetic resonance, *DENSE:* displacement encoding with stimulated echoes, *DL:* deep learning, *LV:* left ventricle, *RV:* right ventricle, *QCD:* quality control driven, *ED:* end diastole, *ES:* end systole.

Image segmentation, which partitions images into anatomically meaningful regions, represents the earliest and most mature application of DL in CMR and is a crucial step in numerous post-processing applications including visualization and quantification. Most commonly, segmentation is used to define epicardial and endocardial borders of the left ventricular (LV) myocardium to calculate myocardial mass and function on cine images [36], [37], [38], [39], to quantify myocardial tissue properties on parametric T1- and T2-mapping [40], [41] and to calculate scar volume on LGE imaging [42], [43], [44] (Fig. 5A-C). DL has also been employed to segment the right ventricle (RV) to assess RV function [37], [45], the left and right atria to calculate volumes and surface areas [37], [46], and the great arteries to measure flow velocity [47] and aortic distensibility [48]. A popular DL architecture for segmentation tasks is the U-Net [49]—an encoder-decoder convolutional neural networks (CNN) with skip connections, and variants, such as the 3D U-Net [50] and nnU-Net [51], have also been employed. More recently, Vision Transformers [52], [53], [54] using an alternative architecture to the CNN have demonstrated potentially superior performance in CMR segmentation.

#### 2.1.3 Image registration

**Fig. 6:**
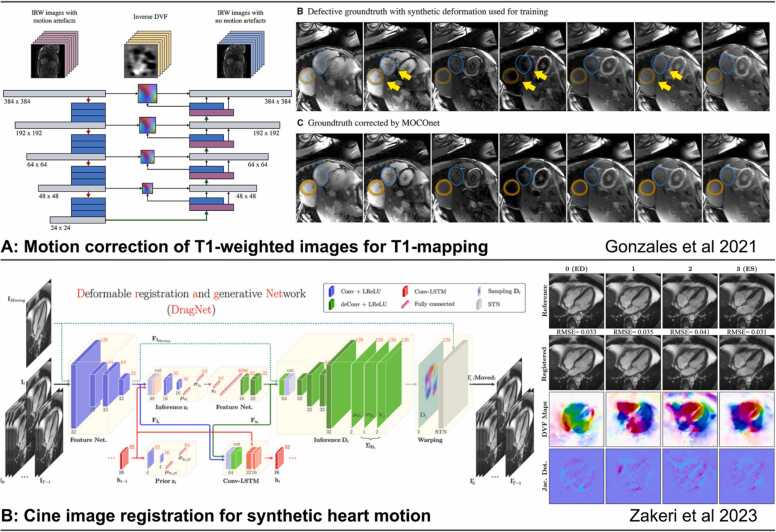
AI in CMR image registration. (A) Supervised learning for T1-mapping motion correction [59]. (B) Unsupervised learning-based deformable registration of cine for generating synthetic cine sequences from a single frame [61] *AI*: artificial intelligence, *CMR*: cardiovascular magnetic resonance, *IRW*: inversion recovery weighted, *DVF*: displacement vector field, *MOCOnet*: motion correction network, *STN*: Spatial transformer network, *LReLU*: Leaky Rectified Linear Unit, *LSTM*: Long Short-Term Memory.

Image registration is a process that aligns two or more images of the same object. Cardiac image registration is a complex problem due to non-rigid motion, mixed motion patterns caused by both intrinsic heart motion and breathing, limited anatomical landmarks, and variations in spatial and temporal resolutions and contrast between images. DL-based registration methods have emerged as a promising alternative to conventional methods due to their ability to handle complex image features and adapt to different image contrasts and registration scenarios [57]. DL image registration for CMR can utilize supervised learning [58], [59] (Fig. 6A). It can also utilize unsupervised learning using generative models [60], [61] guided by similarity in intensities between images [62], [63] (Fig. 6B). Image registration is a fundamental step in cardiac image processing, and the result can facilitate or be combined with further analysis, such as image segmentation [58], motion correction [64], and motion estimation [65]. Compared to traditional methods, DL registration methods are typically much more inference-efficient, providing the possibility of real-time guidance and inline processing.

#### 2.1.4 Landmark detection

Localization of landmarks and anatomical structures is a common pre-processing step for CMR view planning and image analysis. DL has been applied to detect key landmarks and use them to prescribe imaging planes [66]. This can automate CMR pilot imaging and view planning [67], reduce human intervention in imaging, and reduce the overall exam time. Landmark localization in CMR post-processing commonly includes the detection of RV insertion points on short-axis images to assign American Heart Association (AHA) segments [36], and the tracking of the mitral valve plane and apical points on long-axis images [56] (Fig. 5C and D). These networks usually take one of two forms, either an image-to-image translation model to output the probability map of landmark locations [56], [68] or an image-to-vector regression model to output the predicted coordinates of the landmarks on the images [69].

#### 2.1.5 Quality control

QC of images should occur before or be integrated into the image analysis pipeline, as insufficient image quality can result in image segmentation and other errors and may compromise diagnostic and prognostic accuracy. Also, QC applied during a CMR exam could facilitate image reacquisition to replace low-quality images [70]. Currently, in clinical practice, image quality assessment is performed visually; however, this practice will likely change in the context of fully-automated DL-based image analysis pipelines for CMR.

Pre-analysis QC can address multiple quality issues. Multiple studies have developed QC methods to detect motion-related artifacts like those from mis-triggering, arrhythmias, and inconsistent breath-holding using a variety of AI methods [71], [72], [73], [74], [75], [76], [77], [78]. Suboptimal image contrast can also be identified [70], [71], [72]. In addition, DL has been used to detect improper slice orientation, such as the presence of the LV outflow tract in a four-chamber view, foreshortening of the apex, and the absence of valves in the three-chamber view [73], [76]. AI methods can also detect incomplete coverage of the LV in short-axis stacks using methods, such as Fisher-discriminative 3D CNNs [79] and hybrid decision forests [71], [72]. It has also been shown that a CNN can mimic the image quality assessment of an expert using a numerical quality scale [70].

Post-analysis QC has mainly focused on the evaluation of the output of segmentation models.

QC-driven segmentation frameworks attempt to infer well-known validation metrics, such as the Dice score [38], [80], the Hausdorff distance, or uncertainty estimates by using ensemble DL models [38], [78], multi-task learning [77], a multi-view network [81], or multi-level two-dimensional (2D) and three-dimensional (3D) DL-based methods [82]. More studies use a QC framework to detect CMR segmentation failures using descriptors in a random forest classifier [83], using the approach of Reverse Classification Accuracy [84], and combining uncertainly maps with DL models [85], [86]. Other post-analysis QC can be performed by detecting abnormalities in the computed LV/RV volumes and strain curves using a support vector machine [73].

#### 2.1.6 Classification (diagnosis)

**Fig. 7:**
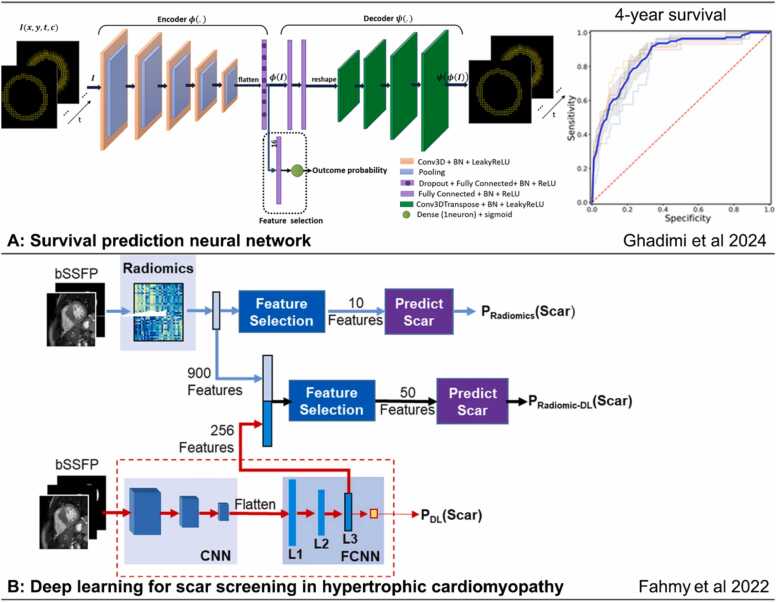
Classification AI models for diagnosis and prognosis. (A) A recent survival prediction neural network. The input is the DENSE displacement through time, the outputs are the DENSE displacement reconstructed from latent features, and the probability of 4-year survival after CRT. ROC curves are for 4-year survival prediction [108]. (B) A radiomics and deep-learning approach for scar screening in hypertrophic cardiomyopathy [109]. *AI*: artificial intelligence, *DENSE*: displacement encoding with stimulated echoes, *bSSFP*: balanced steady state precession, *FCNN*: fully connected neural network, *BN*: batch normalization, *ReLU*: Rectified linear unit, *CNN*: Convolutional neural netwrok, *CRT*: cardiac resynchronization therapy, *ROC*: receiver operating characteristic.

Classification and regression AI models allow for automated diagnosis, prognosis, therapy response prediction, and risk stratification. These algorithms may take parameters derived from image pre-processing and quantification steps, or directly process images or chamber volumes, automatically extracting pertinent features to make predictions. An exemplar DL model using quantitative displacement CMR to predict survival is given in Fig. 7A. Multi-sequence CMR contains complementary information regarding myocardial tissue properties and heart function. Many AI models have been developed utilizing multiple sequences for diagnosis and outcome prediction for a variety of heart conditions; a non-exhaustive list of studies is summarized in Table 1. Additionally, AI has been used to identify ECG phenotypes, such as in hypertrophic cardiomyopathy (HCM) [87], [88] and MI [89], and these combined with CMR and clinical variables, such as patient characteristics and laboratory data, can improve diagnostic accuracy and confidence [90].

**Table 1:** Examples of studies combining multi-sequence CMR for diagnosis or outcome prediction of various heart conditions.

	Cine	T1 map	T2 map	Perfusion	ECV	LGE	Disease or conditions
Khozeimeh et al. [91]	✓		✓	✓		✓	CAD
Pezel et al. [90]				✓		✓	CAD
Khozeimeh et al. [91]	✓		✓	✓		✓	CAD
Shu et al. [92]	✓					✓	DCM
Shi et al. [93]		✓			✓		HCM
Agibetov et al. [94]	✓	✓				✓	Cardiac amyloidosis
Martini et al. [95]	✓					✓	Cardiac amyloidosis
Sharifrazi et al. [96]	✓	✓	✓			✓	Myocarditis
Moravvej et al. [97]	✓	✓	✓	✓		✓	Myocarditis
Ghareeb et al. [98]	✓	✓	✓			✓	Myocarditis
Eichhorn et al. [99]	✓	✓	✓		✓	✓	Myocarditis
Cau et al. [100]	✓	✓	✓			✓	Takotsubo
Mannil et al. [101]			✓			✓	Takotsubo
Dykstra et al. [102]	✓					✓	Atrial fibrillation
Cornhill et al. [103]	✓					✓	HF hospitalization
Bivona et al. [104]	✓					✓	Resynchronization
Kwak et al. [105]	✓				✓	✓	Aortic stenosis
Lu et al. [106]	✓					✓	Sarcoidosis
Okada et al. [107]	✓					✓	Sarcoidosis

*ECV* extracellular volume, *LGE* late gadolinium enhancement, *CAD* coronary artery disease, *DCM* dilated cardiomyopathy, *HCM* hypertrophic cardiomyopathy, *HF* heart failure.

Further, the ability of AI techniques to handle high-dimensional data has led to the development of radiomics, a novel field in which digital medical images are converted into mineable high-dimensional data by extracting a large number of quantitative features [109], [110], [111], [112] (Fig. 7B). Within the field of CMR radiomics, texture analysis allows for analysis and classification of medical images based on underlying tissue inhomogeneities [11], [93], [113], [114].

### 2.2 AI applications in CMR sequences

The previously described AI methods have been applied to a wide range of CMR sequences (Fig. 3). In this section, we summarize several of these studies.

#### 2.2.1 Cardiac cine imaging

CMR cine imaging provides accurate and reproducible measurements of cardiac anatomy and function. Cine images are routinely collected using ECG-gated segmented acquisitions during multiple breath-holds. For patients who exhibit difficulty with breath-holding or with an irregular cardiac rhythm, real-time cine imaging can be acquired, albeit with reduced spatial and temporal resolution.

Accelerated acquisition and advanced reconstruction methods can increase temporal and spatial resolution or reduce the scan time for both segmented and real-time cine imaging. Diverse DL methods have been applied in this context, including methods using image-domain-based artifact reduction [25], [29], [33], [115], [116], hybrid techniques with direct mapping from k-space to the image domain [117], [118], [119], [120], and super-resolution reconstruction from low-resolution inputs [121]. Some proposed methods have achieved 12-fold and 13-fold undersampling for accelerated acquisition. Many of the studies to date were limited to retrospective undersampling of the data (usually using a single coil) [29], [33], [115], [117], [120], healthy subjects [29], [115], [116], and image quality evaluation using non-clinical quantitative metrics only [29], [115]. Two of the aforementioned studies involved testing in prospectively undersampled data from patients [33], [118].

Detection of landmarks and anatomies is an important pre-processing step for the automated analysis of cine imaging. For example, localizing the structure of interest (e.g., the LV) can improve the confidence and accuracy of anatomical segmentation [50]. Detecting the mitral valve plane and the LV apex on the long-axis cine image determines the orientation and length of the ventricle [56]. Tracking of these landmarks through time on long-axis cine images provides key metrics of systolic and diastolic function [69]. Segmentation of the LV allows automated extraction of anatomical parameters, such as LV myocardial mass and wall thickness, and functional parameters, such as LV ejection fraction (LVEF). Segmentation of the RV [45], left atrium (LA) [122], and great arteries [123] on cine images has also been studied, with similar tasks of quantifying anatomical and functional parameters for those chambers. As the most intensively studied sequence in the area of AI for CMR, automated reporting on cine imaging may soon become available as inline methods on scanner platforms [56].

A number of studies have used DL-based automated segmentation of cine images for outcome prediction. For example, one study showed that DL-based segmentation to compute LVEF is as effective as conventional analysis of cine images for predicting major adverse cardiac events in MI patients [124]. Another study evaluated the performance of a DL-based multi-source model (trained using clinical and extracted motion features) for survival prediction and risk stratification in patients with heart failure and reduced ejection fraction (HFrEF). The proposed model could independently predict the outcome of patients with HFrEF better than conventional methods [125]. Another example used DL-based segmentation of cine images along with motion tracking of the RV to improve survival prognostication in patients with pulmonary hypertension [126]. Additionally, DL-based automated segmentation of cine images has been shown to be successful for prognostication in patients with tetralogy of Fallot [125], [127], [128].

In the area of diagnosis, DL was used to segment the LV and extract motion features from cine CMR to detect chronic MI [129]. Also, recently multilinear subspace learning was employed to identify and learn diagnostic features in patients with suspected pulmonary arterial hypertension (PAH) without the need for manual segmentation [130]. In this study, learned features were visualized in feature maps, which confirmed some known diagnostic features and identified other, potentially new, diagnostic features for PAH.

#### 2.2.2 Strain

**Fig. 8:**
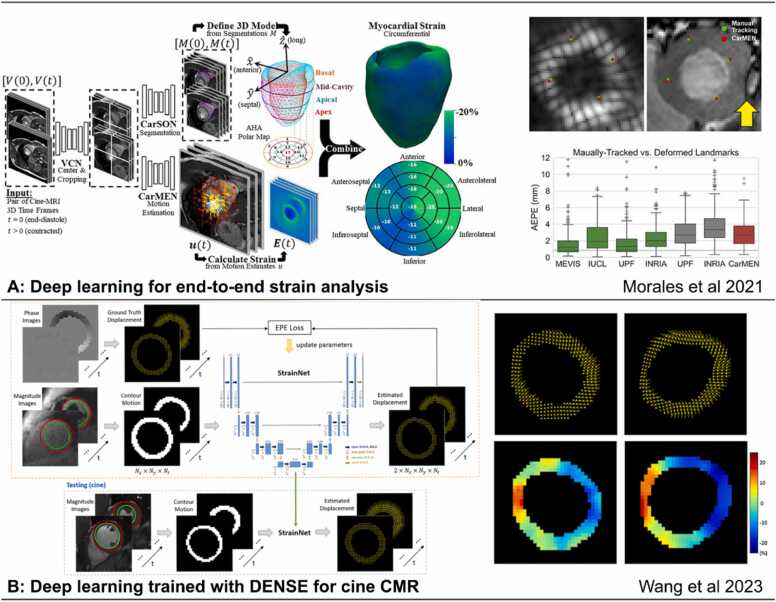
AI in feature tracking and strain analysis. (A) An end-to-end automatic strain analysis from cine MRI to quantitatively characterize cardiac mechanics with deep learning-based cine segmentation and feature tracking [63]. (B) A convolutional neural network trained with DENSE data for displacement and strain estimation applied to cine MRI showed better performance than commercial feature tracking, reprinted with permission [135]. *3D*: three-dimensional, *AI*: artificial intelligence, *CMR*: cardiovascular magnetic resonance, *DENSE*: displacement encoding with stimulated echoes, *MRI*: magnetic resonance imaging, *AEPE*: average end point error, *MEVIS*: Fraunhofer *MEVISIUCL*: Imperial College London - University College London, *UPF*: Universitat Pompeu Fabra, *INRIA*: Inria-Asclepios project (The National Institute for Research in Digital Science and Technology), *CarMEN*: cardiac motion estimation network, *CarSON*: cardiac segmentation network, *EPE*: end point error, *VCN*: ventricular centering network.

Strain and strain-rate imaging quantify deformation of myocardial muscle and provide a quantitative assessment of myocardial function with greater sensitivity to LV dysfunction than LVEF. DL-based automatic or semi-automatic segmentation of the LV, RV, and LA facilitates the efficient use of feature tracking or other methods to compute strain for each of these chambers [100], [131], [132], [133]. Indeed, DL-facilitated fully-automated feature-tracking strain from LV cine images has achieved prognostic accuracy equivalent to that of manual-based segmentation for acute MI as shown in a study of over 1000 patients [131]. Similarly, in light-chain amyloidosis, DL-facilitated LA strain was shown to provide independent and additive prognostic value for all-cause mortality [133]. In addition to segmentation, DL can further improve the diagnosis based on strain. A fully-connected neural network taking strain features as the input has outperformed conventional methods to discriminate HCM from its mimic states, namely cardiac amyloidosis, Anderson–Fabry disease, and hypertensive cardiomyopathy [134]. In addition to feature tracking analysis of cine CMR, unsupervised DL has been explored to compute displacement and strain from cine CMR, leading to the DeepStrain method [63] (Fig. 8A). Using supervised learning, displacement encoding with stimulated echoes (DENSE) data have been employed to develop StrainNet [135], and velocity-encoded data have been used to develop synthetic strain [136], both of which can be applied for strain analysis of cine CMR (Fig. 8B).

DL for strain also extends beyond the analysis of cine imaging to strain-dedicated CMR sequences. For example, DL-based methods to analyze tagged CMR images have been shown to be superior to harmonic phase analysis with regard to tag tracking accuracy and inference efficiency [62]. For the analysis of DENSE CMR, DL for LV segmentation and phase unwrapping provides fully-automated, highly accurate and reproducible results for both global and segmental circumferential strain [36].

#### 2.2.3 Late gadolinium enhancement

LGE is an established and validated CMR technique to distinguish myocardial fibrosis and injury from normal myocardium [137], [138]. Routine clinical use involves 2D acquisitions and is limited by spatial resolution, long scan times, and the requirement for breath-holds [139]. Novel frameworks enabling 3D acquisitions have been proposed and DL has been applied to accelerate LGE reconstruction [140]. DL-based noise reduction has also been applied to improve the image quality of fast, low-resolution LGE images [141].

In current clinical practice, LGE scar reporting usually relies on visual assessment by experienced clinicians. In research, scar quantification is currently based on manual delineation of myocardial borders and regions of enhancement, followed by thresholding techniques. For automated quantification, landmark localization [56] and LV segmentation [142], [143] applied to LGE images can be performed with DL. Subsequently, scar/fibrosis segmentation is essential for quantifying scar size and volume fraction [7], [143], [144], [145]. Automated segmentation has been applied to LGE for LV myocardium and ischemic scar segmentation [145], [146], while LGE scar segmentation and quantification for non-ischemic heart disease remain challenging due to the complex patterns of myocardial fibrosis and variations in gadolinium kinetics.

Quantification and segmentation of LGE images can be assisted by routine cine CMR, as cine provides complementary features and better-defined myocardial borders compared to LGE. For example, registration of LGE and cine CMR is beneficial for improving localization and quantification of infarcted regions [147]. Further, joint approaches for image registration and segmentation of LGE and cine offer better performance than segmentation of LGE images alone [148].

#### 2.2.4 Parametric T1 and T2 mapping

Parametric quantitative mapping measures the relaxation times of tissue protons and reflects physical tissue composition [149]. These methods typically require the acquisition of a series of images sampled at various inversion times or echo times and/or utilize preparation modules to develop contrast. Fitting the series of images to a corresponding signal model, in a pixel-wise manner, enables the generation of a quantitative map of T1 or T2 tissue relaxation expressed in units of time (e.g., milliseconds). Current clinical protocols entail relatively lengthy 2D acquisitions with moderate spatial resolution that require breath-holding. In the case of accelerated acquisitions, conventional model-fitting reconstruction techniques are susceptible to aliasing artifacts and noise [150].

A DL-based network that allows sharing information across pixels has recently been applied to T1 mapping which reduced image noise by performing spatial and temporal regularization [150]. Additionally, a neural network, termed Robust artificial-neural-networks for k-space interpolation, which applies non-linear physics-based k-space estimation from undersampled k-space data, has been utilized to recover accelerated SAturation Pulse Prepared Heart rate independent Inversion-REcovery sequence T1 maps [28]. The technique can be regarded as a DL extension of the Generalized Autocalibrating Partially Parallel Acquisition (GRAPPA). A unique aspect of this method is that it is scan-specific; i.e., the network is trained from the center k-space lines of the same scan, therefore obviating the requirement of large training sets. This method was evaluated in retrospectively undersampled data from healthy subjects with quantitative metrics and outperformed traditional GRAPPA reconstruction particularly at five-fold acceleration [28].

Segmentation of LV myocardium is a necessary step for measurement of T1 and T2 values, which can be automated with DL [38]. Patient movement during a CMR scan can cause changes in heart position between the raw images, leading to motion artifacts in the resulting maps. Motion correction can be performed using DL techniques [59], [151] to register the raw images and restore precise T1 and T2 values and parametric maps. Deep generative models have also been developed to enhance T1-mapping signals, combing them with cine for more robust and informative scar imaging in the presentation of “virtual native enhancement” (VNE) [152], [153], [154] that resembles “virtual LGE”, holding promise for fast and gadolinium-free myocardial tissue characterization with further technical development.

#### 2.2.5 Multiparametric quantitative MRI

Simultaneous multiparametric quantitative MRI, where several parameters of interest are obtained from a single scan, has recently gained attention to preclude confounding of the different parameters and achieve a shorter scan time. Several models have been investigated including magnetic resonance fingerprinting (MRF) [155], multitasking [156], and others [22], [157], [158], [159], [160]. Particular hurdles for cardiac MRF include long acquisition and reconstruction times and the requirement for scan-specific dictionary generation based on the patient- and scan-specific heart rhythm.

To overcome some of these limitations, a combination of DL-based denoising and low-rank modeling has been applied to accelerate the MRF acquisition and shorten the breath-hold duration [161]. Furthermore, a fully-connected neural network to directly quantify T1 and T2 from MRF images, bypassing dictionary generation and pattern matching and reducing computation time and memory requirements, has been proposed [35]. Cardiac multitasking has been applied using a low-rank tensor approach with two spatial dimensions and three time-dimensions (cardiac phase, respiratory phase, and inversion time), to enable non-ECG-gated, free-breathing dynamic imaging, and was demonstrated for T1-mapping. Validation in healthy subjects demonstrated similar-quality images and T1 maps to conventional iterative methods, while reducing the reconstruction time by greater than 3000 fold [162].

#### 2.2.6 3D whole-heart imaging

3D whole-heart imaging is an integral part of anatomical imaging in cardiac disease and recent advances are promising for the assessment of coronary arteries using CMR [31]. Nevertheless, long scan times associated with higher spatial resolution and concurrent motion artifacts hinder wider clinical usage. Advances in DL-based reconstruction methods have been investigated to overcome those limitations. The respective algorithms can be divided into three main categories [27]: (1) algorithms that apply non-linear physics-based k-space estimation from acquired k-space data [163], (2) end-to-end data-to-image techniques, where the network parameters are trained to recover the images directly from undersampled k-space data [27], [30], and (3) an end-to-end network that recovers motion fields between highly undersampled respiratory-resolved images that are utilized for motion-corrected reconstruction [164]. Further advances include approaches that achieve super-resolution reconstruction from rapidly acquired low-resolution data [31], [165]. The aforementioned techniques have been tested on healthy subjects [163] and patient cohorts against clinical coronary or anatomical 3D whole-heart imaging with satisfactory quantitative and qualitative image quality metrics, providing significantly shorter acquisition time. It is worth mentioning that DL may be able to leverage the high-resolution data of CT angiography and transfer the knowledge to CMR to optimize the contrast and resolution of CMR angiography [31].

Current 3D whole-heart frameworks use diaphragm-based navigation, which limits respiratory scan efficiency [166]. Image-navigator and self-navigated [167] techniques have been proposed to account for the complex non-rigid respiratory-induced cardiac motion to achieve high-resolution 3D isotropic scans. However, non-rigid motion estimation/correction is frequently dependent on image registration [168], [169] and laborious image reconstruction techniques. To address these limitations, DL-based estimation of non-rigid cardiac motion has been proposed and validated. A fundamental network enabled a 20-fold speed up in the non-rigid motion estimation step, reducing computation time of the image registration step [170]. The pipeline was further extended to the final motion-corrected reconstruction, reducing the total computational time by 50-fold [171]. Automated image quality assessment for 3D whole-heart imaging has been implemented. It is proposed to estimate image quality with good agreement with respect to human expert reading and may help identify the optimal reconstruction framework or define termination criteria of an iterative reconstruction process [70].

#### 2.2.7 2D phase-contrast MRI

Phase-contrast MRI is an integral component of CMR protocols enabling quantification of blood flow in the great vessels, estimation of valvular regurgitation, and internal validation of the ventricular stroke volumes [172]. Conventional phase-contrast MRI methods use ECG synchronization and, frequently, strategies of k-space segmentation to reduce acquisition time to a breath-hold duration. Free-breathing accelerated acquisitions are also clinically relevant for patients with difficulty in breath-holding (for example, for children and patients with dyspnea) and for real-time applications, such as exercise stress CMR [34].

DL-based reconstruction methods have been applied to recover images acquired using undersampled radial [34] and spiral trajectories [173]. Both methods have been trained using synthetic breath-held and ECG-gated datasets and evaluated in prospectively acquired free-breathing undersampled phase-contrast images [34], [173]. Qualitative and quantitative metrics and quantifiable hemodynamic parameters demonstrated satisfactory agreement with conventional acquisition and reconstruction techniques with acquisition times that were 28-fold and 18-fold faster, respectively.

#### 2.2.8 4D flow

Four-dimensional (4D) flow MRI is an emerging technique where 3D blood velocity over time can be captured with full volumetric coverage in a single scan. Challenges for further improvement of 4D flow include velocity aliasing due to suboptimal velocity encoding, low spatiotemporal resolution, and long reconstruction times [174].

Approaches to optimize 4D flow acquisition and reconstruction methods have been investigated. DL-based velocity antialiasing has been tested in healthy-volunteer datasets, demonstrating moderate to excellent agreement to ground truth [175]. Furthermore, a physics-based model has been applied to ECG-gated and breath-hold datasets, achieving reconstruction in under 1 min, which was 30 times faster than state-of-the-art compressed-sensing methods [176].

DL-based segmentation techniques have been applied to 4D flow to delineate the vessel lumen to facilitate calculation of mean velocities and aortic flow quantification [177]. Segmentation can be performed on 2D images [47], bSSFP cine images (with interpolation onto flow CMR) [178], or 3D phase-contrast MR angiograms [179]. Fully-automated 4D flow segmentation remains challenging due to the low blood-tissue contrast in magnitude images, insufficient phase-contrast signal with low velocities, and the requirement for 3D analysis [177].

#### 2.2.9 Perfusion MRI

CMR perfusion imaging is a non-invasive test to assess myocardial blood flow and ischemia. Myocardial perfusion is assessed by imaging the LV myocardium during the first pass of a contrast agent bolus. To detect ischemia, perfusion imaging is performed both at rest and at stress, where stress imaging utilizes vasodilation by a pharmacological agent such as adenosine. High temporal resolution is required, often compromising spatial resolution and coverage. Accelerated imaging techniques are required to optimize the balance between those parameters.

Several approaches for DL-based image enhancement networks have been proposed. Those have been trained in a supervised manner using conventional compressed-sensing reconstruction outputs as reference images [180], [32]. Evaluation included both quantitative and qualitative image quality scores in healthy subjects [32], [181] and in patients [180], demonstrating comparable or superior image quality scores compared to compressed sensing with a much shorter reconstruction time. A physics-guided neural network, based on a signal intensity informed multi-coil encoding operator, has been recently proposed to capture the signal intensity variations across time-frames, allowing highly-accelerated simultaneous multislice myocardial perfusion cardiac MRI [181]. This physics-guided DL framework enabled self-supervised training from undersampled k-space data only, obviating the requirement for reference images and large training datasets. The physics-guided DL framework outperformed multiple regularized reconstructions, demonstrating improved image quality, and reduced noise amplification and aliasing.

Myocardial perfusion analysis involves the delineation of a time series of images to compute myocardial perfusion reserve and present it using the format of the AHA model, which is time-consuming when using conventional methods. DL has been proposed to automate the process by localizing the RV insertion points and LV in a time series of perfusion images [182], [183], [184]. Furthermore, DL algorithms have been successfully applied to the segmentation of LV cavity and myocardium, where the quantification of myocardial blood flow and perfusion reserve parameters produced outputs comparable to manual analysis [182]. In another study, the automatic segmentation and quantification of perfusion mapping provide a strong, independent predictor of adverse cardiovascular outcomes [185].

#### 2.2.10 Protocol planning and efficiency

In addition to the role of AI for individual sequences, AI can improve the efficiency of the workflow by automating the CMR protocol planning. For example, by performing landmark detection or regression algorithms on short-axis views, long-axis views, or additional imaging, DL can determine the orientation of the LV for automated prescription of image planes. An EasyScan technique was reported to offer clinically acceptable planes on par with expert CMR technologists [186]. Another study has demonstrated that by predicting landmarks on multiple images and views, DL can prescribe common CMR view planes similar to those marked by a radiologist or those prescribed by a technologist at the time of image acquisition [187]. Further, in CMR imaging, careful shimming is required to establish a homogeneous B0 field and on-resonance center frequency around the heart, especially for bSSFP sequences. AI-based shimming technique can automatically adjust the field, leading to increased signal-to-noise ratio and contrast [186]. These, together with post-processing DL techniques for common CMR sequences, can be integrated into typical CMR workflows, leading to “one-click” CMR scanning with reduced input demands on MR technologists and reduced scanning time.

## 3 Roadmap to translate advances in AI CMR research to routine clinical use

### 3.1 Need for high-quality and representative datasets

**Fig. 9:**
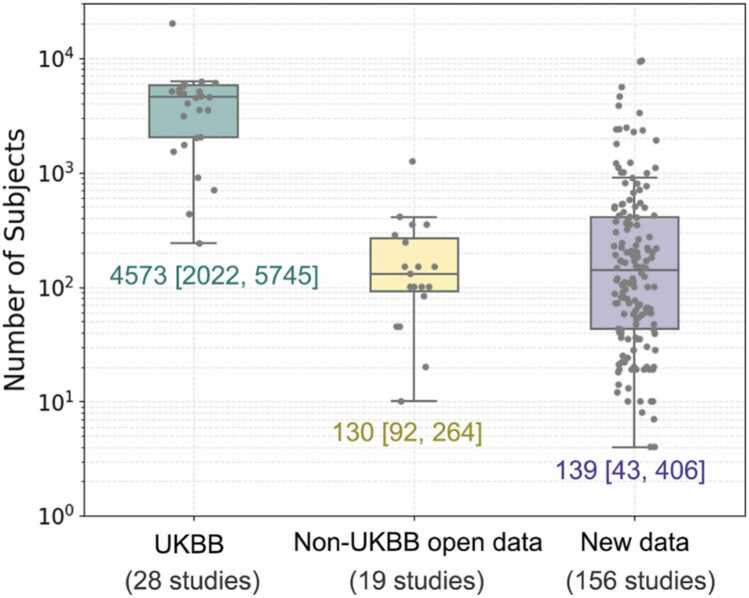
Distribution of number of subjects by dataset source in 203 AI CMR papers. UK Biobank (UKBB), Non-UKBB Open Data, and New Data are from 28, 19, and 156 papers, with median of 4573 (Q1: 2022, Q3: 5745), 120 (Q1: 92, Q3: 264), and 139 (Q1: 43, Q3: 406) subjects, respectively.

Training and evaluating an AI prediction model requires reliable data at sufficient scale and diversity. An appropriate sample size is determined by the expected effect size and the classification accuracy of the model [188]. Models with a large number of tunable parameters may be overfit on small samples such that predictions do not generalize to new data. However, it may not be feasible or affordable to curate very large annotated clinical datasets for every use case. A technical proof-of-concept study may utilize a smaller dataset to show feasibility of a new method, whereas an AI model intended for widespread clinical use would require a larger and more comprehensive dataset for both development and validation. Increasing the dimensionality of the data, from 2D to 2D with a temporal dimension (2D+t), 3D or 3D+t, may also make the data relatively sparse and at risk of overfitting. Fig. 9 summarizes the dataset distribution of 203 CMR-related AI papers, of which 47 utilized open access datasets, including from the UK Biobank [189], [190], ACDC [191], M&Ms [192], HVSMR [193], MS-CMRSeg challenge [194], [195], SunnyBrook Cardiac Data [196], Harvard radial raw data [120], and XCAT phantom data [197]. In the studies utilizing UK Biobank data (28 studies), open access data other than UK Biobank (19 studies), and newly acquired data (156 studies), the median (25th, 75th percentiles) of subject numbers are 4573 (2022, 5745), 130 (92,264), and 139 (43,406), respectively. The average training to testing dataset ratio reported from 124 papers is 5.49:1.

Diverse and representative data are especially important in the translational stage of AI models. Many AI models have shown better performance than human operators on specific test sets [198], [199] but may generalize poorly to other settings that have distribution shifts [200], which hinders their widespread clinical use. Data quality is similarly important. Data annotation requires intensive manual analysis by experienced image analysts and is prone to error. Novel unsupervised learning may obviate the need for laborious data annotation, but large, good quality CMR datasets are essential.

Obtaining representative datasets is challenging due to the scarcity of properly annotated data, the shortage of data covering all relevant cardiovascular diseases, and the presence of artifacts that might result in low-quality training datasets. A useful AI solution to overcome this challenge is data augmentation, where deep generative models have been proposed for synthesis of large, high-quality medical images with variability in anatomical representation and appearance, comparable to real counterparts. The primary approaches in image synthesis are: i) mask-to-image synthesis, where segmentation masks are mapped to corresponding images (the inverse of image segmentation) [201], [202], [203], [204], ii) image-to-image inference [205], and iii) regression models [201], [206], [207]. For example, augmenting real data with synthetic data during training has been shown to improve the performance of cine image segmentation [201], [204]. Similarly, augmenting LGE with VNE modality in AI development improved the accuracy and reliability of LGE segmentation [44].

Nevertheless, for clinical application, DL models should be thoroughly validated on real data that are diverse in terms of gender, race, environment, body habitus, types of disease, sites, MRI vendors, and platforms to ensure model robustness and generalizability. While cross-validation techniques are useful for assessing internal validity, independent external validation on the intended population will assess transportability of the model. Even external validation is not a one-off process before clinical implementation and may require further cycles of recalibration if the model is sensitive to small population differences [208]. Regulatory agencies have also identified evidence for other AI tools in medical imaging that include impact on clinical decision-making, diagnostic accuracy alongside physician review and patient perceptions [209]. Guiding principles on the safe and equitable deployment of AI algorithms may include effective governance oversight, multi-disciplinary evaluation, continuous surveillance, and incorporation of consensus guidelines on the use of AI tools [210].

CMR datasets that are discoverable and open access can accelerate advancements across the entire field. Organizations, such as the SCMR and the National Institute for Health, should encourage data sharing for training and testing AI algorithms, ensuring that data are findable, accessible, interoperable, and reusable [211]. In addition, they should promote the establishment of multi-site datasets accounting for variabilities between sites, with ethics and data governance policies in place. It is also important to acknowledge that non-ideal but unique or distinctive datasets, with their inherent limitations, can make substantial contributions to the field, potentially offering opportunities to train unique AI models and/or demonstrate proof of concept in new and innovative directions.

### 3.2 Need for guidelines for reporting AI CMR research

To promote quality, improve reproducibility, and increase adoption, there is a need for guidelines for reporting AI-based research. Such guidelines will help the community better understand and assess study findings and their potential clinical impact. Several guidelines for AI in medicine and medical imaging have been proposed over the last few years or are currently under development. For example, CLAIM (Checklist for Artificial Intelligence in Medical Imaging) [212], a guideline for authors and reviewers, suggested a list of information that manuscripts should provide related to models, training procedure, datasets, etc. Similarly, MINimum Information for Medical AI Reporting [213] specified the minimum information that authors of manuscripts should provide in terms of study population, data demographics, model architecture, and model evaluation. CONSORT-AI (Consolidated Standards of Reporting Trials) and SPIRIT-AI (Standard Protocol Items: Recommendations for Interventional Trials) [214], [215] were extensions of CONSORT and SPIRIT and provided guidelines for reporting randomized trials involving AI-based methods. Also, FUTURE-AI [215] provided a list of best practices based on six principles: Fairness, Universality, Traceability, Usability, Robustness and Explainability (FUTURE) that should guide AI-based research to provide trustworthy solutions. Last, Transparent Reporting of a multivariable prediction model for Individual Prognosis Or Diagnosis - Artificial Intelligence and Prediction model Risk Of Bias Assessment Tool - Artificial Intelligence [216] (extensions of TRIPOD and PROBAST guidelines) are currently under development as guidelines for reporting and risk of bias assessment of clinical prediction models using AI. The CMR community may adopt these guidelines (individual or a combination of these) [217], [218] or extend and modify them to promote high-quality AI-based CMR research. A summary of the most relevant recommendations from these publications is provided in Table 2, this summary follows but also complements the CLAIM recommendations.

**Table 2:** Summary of best practices for authors and reviewers for AI in medical imaging.

Section	Best practices
Manuscript title and abstract	Clearly indicate the AI methodology used and provide a structured summary of the study’s design, methods, results, and conclusions.
Introduction	Provide the scientific and clinical background of the AI approach employed and state how the proposed approach will help addressing a significant clinical or scientific issue.
Methods and results	• Provide details of the study design, including prospective or retrospective nature, data sources, pre-processing steps, and ground truth definitions. • Provide a comprehensive description of the model’s structure, training procedures (data partitions), and performance metrics. • Use appropriate metrics to evaluate model performance and report results with statistical significance. • Include validation or testing on external data. • Consider including experiments to address potential bias. • Consider including experiments to address generalization. • Consider sharing open-source code to enable reproducibility studies.
Discussion	Summarize results, discuss limitations including potential bias and generalizability issues, implications for practice, and future directions.

This summary follows but also complements the CLAIM recommendations.

*AI* artificial intelligence, *CLAIM* Checklist for Artificial Intelligence in Medical Imaging.

### 3.3 Considerations around clinical deployment

AI-based methods are to a large extent data-driven, and hence may unintentionally replicate biases that are hidden in those data [219]. Lack of diversity in the training datasets on gender, race, age, ethnicity, weight, height, and social disparities with regards to access to health care (particularly at academic research centers) might lead to suboptimal performance. Several approaches have been proposed to address this matter that span from the pre-training, training, and post-training stages of data, including comparing standard performance metrics across different sub-groups, and the employment of the fairness-specific criteria to audit for the presence of bias in a given model [220]. From a regulatory standpoint, several strategies have been developed to address the issue of biased data in AI systems. The STANDING Together initiative (standards for data diversity, inclusivity, and generalizability), launched in September 2022, aims to develop recommendations for the composition (who is represented) and reporting (how they are represented) of datasets utilized in medical AI systems. The panel is comprised of patients and the public, clinicians and academic researchers across biomedical, computational, and social sciences, industry experts, regulators, and policy-makers, and the final recommendations are rooted in an 18-month program of systematic reviews, surveys, in-depth interviews, and a modified Delphi study [221].

AI systems raise concerns about transparency and accountability, as they are programmed to “learn” a model from a large set of data, without providing a rationale for the outcome [222].

Before deploying a DL system in the critical infrastructure of medical imaging, validation of the decision pathway and the ground-truth knowledge should be provided. From a technical perspective, various metrics and visualizations are available for evaluating the technical performance of AI algorithms [216]. Saliency methods, although widely used in medical studies for model interpretation and localization, have been scarcely applied in AI techniques for CMR, and their utility in non-CMR applications is debated [223], [224]. To address trustworthiness in AI comprehensively, explainable AI models (XAI) have been introduced in cardiac imaging [225], which focus on exposing AI models to humans in an interpretable manner [226]. Three levels of evaluation of the outcomes of XAI have been proposed. The first one applies proxies and statistical methods (functionally grounded evaluation), followed by the evaluation by non-clinical evaluators (human-grounded), and lastly by medical experts (application-grounded) [225]. A list of open-source XAI tools currently available can be found in [227]. Furthermore, to promote accountability, several procedures are established and are anticipated to evolve to validate system performance in a tiered manner. A recent systematic review proposed a user-centered research design approach, whereby the model designers actively consider and work closely with the stakeholders (clinicians, patients, technologists, etc), particularly during the design and construction of AI models [228]. Explicit guidelines are published for the evaluation of clinical performance of AI applications [215], [216], [229], [230], [231]. To augment the generalizability of current data-driven AI techniques [232], [233], testing of the algorithms—preferably in multiple sites and conditions in real-world settings—is crucial [199], [234], [229]. Paired and parallel study designs have been proposed to evaluate the benefits of AI in clinical practice [229] and randomized clinical trials remain the gold-standard [229]. Further approaches that incorporate human intervention in the network pipeline, the so-called human in the loop, have also been proposed [10].

Successful AI deployment in clinical practice requires the active involvement of all stakeholders, including patients, academics, clinicians, imaging technicians, hospital administrations, regulatory bodies, and industry. Academics and clinical associations can aid health care professionals acquire the basic knowledge of AI, to facilitate critical evaluation of datasets, integration within clinical workflows and bias control. A key theme that has to be implemented across the different stakeholders is the requirement for standardized high-quality datasets, to maximize the potential innovations derived and the transparency in how datasets are acquired, to allow better understanding of their context and limitations [235]. This can be achieved by setting standards and guidelines in the entire process of medical image preparation, from the de-identification step to the data annotation step and especially in the data curation step [236]. Multi-disciplinary collaboration is also crucial to promote effective data-sharing models to optimize the model performance, respecting the legal and ethical aspects of the impact of AI adoption [237]. Communication among the stakeholders is important for the continuous appraisal of the applied AI models to foster quality assurance and product improvement.

The application of AI in CMR that involves personal health information also raises concerns about data protection, autonomy, and privacy. In particular, concerns around meaningful consent and effective anonymization and de-identification of data are valid [216] and need to be addressed at a central regulatory and institutional level. All relevant stakeholders should be familiar with the proposed standards. Last, the social and cultural blueprint of AI is currently largely under-studied [238]. An interdisciplinary approach to the application of AI is advisable to expand its clinical potential in a safe, useful, and fair context [239].

### 3.4 Responsibility of the clinician and the need for interdisciplinary teams

Clinicians in the current era are challenged to explore CMR through the lens of AI. Deployment of AI demands from the clinicians to recalibrate their approach to information. However, information/data does not constitute knowledge. Hence, a robust framework to integrate and interpret the data in a meaningful way for patients is required [240].

The processes of curation and anonymization of data and validation of the clinical performance of AI lie with both the clinicians and technical experts and have been covered in previous sections, highlighting the significance of a multi-disciplinary approach [216]. In addition to those, patient’s consent, accurate interpretation of the results, and communication with patients for optimal decision-making lie primarily with the clinician. In this context, two significant issues arise, namely respect for patients’ autonomy and the clinician’s accountability. Patient’s autonomy is honored in the process of informed consent where individuals must be given the opportunity to agree to and make choices between risks they are exposed to [219]. Furthermore, the clinician is accountable for decisions regarding patient management [241]. The accountability stretches above regulations and should encompass the accountability to the ecosystem in which the AI information will be shared (patients, caregivers, community, industry, health care professionals). Thus, if radiologists and cardiologists are to be incorporating AI into daily practice, basic proficiency in AI methodology and understanding both its potential and limitations are required [242], [198], [243] to address those issues. Several approaches have been proposed [242], [244]. Creating the educational resources necessary for an AI curriculum requires the collaboration of multiple national and international societies, such as SCMR, as well as academic radiology and cardiology departments. These educational efforts will need the involvement of and collaboration with technical experts, such as computer scientists, statisticians, and biomedical engineers. Last, the framework of clinical applications based on AI should be laid in rigorous legislation, which clinicians, technical experts, and industry employees should familiarize themselves with. In Europe, the relevant laws (General Data Protection Regulation [GDPR] Article 22) prohibit any decision-making based solely on automatic processing of personal data, precluding in practice the possibility to rely only on the outcome of an algorithm for sensitive decision-making. Furthermore, Article 22 requires the controller to implement suitable measures to safeguard the subject’s rights and freedoms and its legitimate interests, which has to include the right to obtain human intervention. This human intervention has to be qualified, capable of discovering and recovering unfair outcomes or discriminations (European Data Protection Board) [219]. To our knowledge, there is no analogous legislation elsewhere to date.

### 3.5 Industry considerations on AI in CMR

AI presents great opportunities for improved automation [64], [65], [56], data analysis [7], [8], [9], [36], [37], [38], [39], scan time reduction [24], [25], [26], [27], and image quality improvements [30] in MRI and many new products coming from an MRI vendor have an AI component. AI is especially relevant for CMR due to the higher complexity of the exam requiring complex and time-consuming planning, multiple breath-holds and the use of external devices for cardiac and respiratory gating. In addition, practically every single CMR exam is a subject of post-processing and analysis resulting in a quantitative characterization of the cardiac anatomy, function, and tissue properties, which requires fast and reliable segmentation methods. Both the high complexity of CMR scans and the need for data post-processing dictate the demand and trend for automation in CMR, and AI will play a crucial role in realizing these advancements. Such advancements will facilitate the wider dissemination of CMR to low- and middle-income countries [245], benefiting wider populations and promoting equity in access to health care resources globally.

However, there are also several concerns related to the use of AI in MRI scanners. The topics related to data availability, data diversity, and data quality as well as privacy aspects were discussed in detail in previous sections. These are highly relevant in the development of AI-based products, where it is important to ensure generalization and stable performance on a large scale.

There is a growing number of public databases containing CMR data aiming at supporting research activities on AI-based approaches [246]. This allows for more objective comparisons between different network architectures since the performance of an AI model depends both on the architecture and on the data. It also opens the field to research teams with AI expertise but no access to an MR scanner. However, the use of public databases for product development is typically very limited. This is related to several factors including the terms of use, privacy aspects, as well as the quality of the data. Due to the data-dependent performance mentioned above, the gap between research and product development can be much larger for AI models compared to conventional approaches.

The need for acquiring large training datasets may increase the product development time and its costs substantially. If only limited data can be acquired for certain patient groups, this may lead to limiting the product scope beyond these groups. Self-supervised and untrained methods are also seeing increased interest, but they require much longer processing times, which makes their adoption challenging [247], [248], [249]. On the positive side, the risk of reidentification based on image data alone is relatively low for CMR. This is different than in 3D brain imaging, where there is an additional concern of face reconstruction based on the images requiring face removal techniques to protect the participants’ privacy [250]. Nevertheless, caution should be taken to keep the risk of reidentification as low as possible, especially for small local datasets and rare diseases.

Another risk that comes with the data-dependent performance of AI-based approaches is the use of multiple AI-based methods that were developed independently in different stages of the data processing pipeline. For example, an AI-based image reconstruction may change the output of an AI-based image enhancement that was trained on data processed with a different reconstruction. Even seemingly simple modifications in the image reconstruction like computing a magnitude instead of a complex image will have an effect on the performance of subsequent denoising [251]. Similarly, changes in the image reconstruction and enhancement may lead to changes in the image analysis and quantification [252], [253]. The issue of variability in the performance/results of post-processing tools depending on the input data is not new; however, it becomes more acute with the introduction of AI approaches that are more sensitive to modifications in the data processing pipeline. It is especially difficult to predict the outcome of a combination of multiple AI methods that have been independently developed by different vendors. In-depth analysis of potential changes in end, results may be required to address this issue.

It is interesting to speculate about how AI may impact CMR energy needs and its carbon footprint. On one hand, with the growth of data volume, model size, and training infrastructure, developing AI models for CMR will use energy, leading to a negative effect on the environmental footprint. On the other hand, the application of AI methods could shorten CMR exams, leading to decreased energy usage and a positive effect on the environmental footprint. In this way, an analysis of the impact of AI for CMR on the carbon footprint should take a holistic approach [254], by considering both the savings and benefits that it brings to CMR exams, and the energy costs of AI development.

From a regulatory perspective, it is also unclear how the compatibility between different independently developed AI-based devices/techniques should be handled. An even bigger challenge is ensuring the compatibility of multiple AI-based techniques that allow modifications from real-world training.

Another challenge is the deployment of DL models on medical devices. Increasingly complex models (e.g., unrolled reconstruction networks) paired with high-dimensional input data (e.g., high-resolution 3D data, 4D flow, etc.) necessitate high-end hardware accelerators, such as graphics processing units, to ensure acceptable inference times. These must be available either directly within the scanner platform or the infrastructure needs to be in place to off-load computation to an edge or cloud computing facility, which is not yet a common scenario. This may limit widespread availability of advanced applications by excluding systems already in the field and new lower-end systems due to cost.

### 3.6 Regulatory perspectives and considerations related to AI in CMR

For medical devices deployed clinically in the United States (US), the US Food and Drug Administration (FDA) Center for Devices and Radiological Health assures that patients and providers have timely and continued access to safe, effective, and high-quality medical devices. It is important to acknowledge that other regulatory agencies, such as Health Canada, the European Medicines Agency, and the Therapeutic Goods Administration (Australia) will have jurisdiction depending on the regions. While it would be ideal to provide and compare perspectives across regulatory agencies with different jurisdictions, this article is limited to a detailed review and perspective from the FDA as an example.

As of October 2022, over 500 medical devices incorporating AI/ML technology have been granted marketing authorization by the US FDA through a combination of the premarket approval, 510k, and De Novo regulatory pathways [255]. While the majority of these devices are intended for analyzing radiological data, the general approach to evaluating AI/ML-enabled medical devices is the same regardless of medical specialty. An overview of the regulatory considerations for medical imaging AI/ML devices in the US was recently published [256], of which the main points are briefly summarized here.

Data hygiene is perhaps the most fundamental concern in the evaluation of AI/ML-enabled medical devices. A central principle is that the testing dataset should be independent of the training dataset. This generally means that the testing and training datasets should be collected from different patients and at different clinical sites. At the same time, both development and evaluation datasets should be representative of the target population and the evaluation dataset should be of sufficient size to ensure statistical validity. As was pointed out in Section 3.1, scarcity of complete and properly annotated data is a significant hurdle in the development of algorithms, and it can also be a significant hurdle toward approval of a commercial product.

While the specifics of the performance evaluation for a particular device are informed by both the technology of the device as well as its intended use, AI/ML devices in general are evaluated via standalone performance testing and/or clinical studies. Standalone performance testing is a measure of device performance alone with little to no interaction or interpretation from a clinical end user. When a clinical user needs to interact with the device or interpret the device outputs, depending on the risks posed by the device, an assessment of the device in the hands of the end users may also be needed. To ensure generalizability of device outputs and to better understand performance limitations, results of any performance assessment are generally reported both in aggregate as well by sub-groups based on patient characterizes (e.g., age, race, gender, disease type and stage, etc.) and data acquisition characteristics (e.g., data acquisition site, acquisition device, protocol, etc.).

Guidance documents provide the medical device community with insight into current FDA thinking. While no guidance document specific to CMR currently exists, a series of guidance documents developed for radiological imaging-based AI/ML devices discuss premarket submission details [257] and clinical performance assessment [258] of computer-assisted detection devices and may provide useful information for developers of AI/ML-enabled CMR devices. The guidance document related to the technical performance assessment of quantitative imaging in radiological device submissions may also be relevant [259]. A guidance document broadly providing lifecycle management considerations and premarket submission recommendations for AI/ML-enabled device software functions is promised to publish in draft form by the end of fiscal year 2024 [260].

In June of 2022, the National Heart, Lung, and Blood Institute held a workshop entitled “Artificial Intelligence in Cardiovascular Imaging: Translating Science to Patient Care” [261]. While not specific to CMR, the workshop hoped to address many of the same challenges discussed in this manuscript; that is, to identify challenges and opportunities for AI in cardiovascular imaging, focusing on how various stakeholders can support research and development to move AI from promising proofs of concept to robust, generalizable, equitable, scalable, and implementable AI. The workshop identified both policy and technical needs to further advance clinical translation of AI in cardiovascular imaging [261]. Within this context of rapid innovation and regulatory challenges, the mission of FDA’s Office of Science and Engineering Laboratories (OSEL) is to accelerate patient access to innovative, safe, and effective medical devices through best-in-the-world regulatory science. Through the AI/ML program, OSEL scientists seek to address gaps related to limited training and testing data, bias, equity, and generalizability, develop least-burdensome metrics for the performance assessment of AI/ML devices in situations of high uncertainty, develop evaluation metrics for evolving algorithms, and develop approaches for the effective post-market monitoring of AI/ML-enabled medical devices. OSEL develops and shares regulatory science tools (physical phantoms, methods, datasets, computational models, and simulation pipelines) to help advance medical device development and assessment [262].

## 4 Conclusion

In summary, this article reviews the current landscape, challenges, and potential future directions of integrating AI approaches into CMR imaging. It emphasizes the potential for AI to address several challenges faced by CMR in terms of efficiency, accessibility, and manual image analysis and demonstrates the remarkable (and rapid) research progress in AI applications across various AI CMR tasks and diverse CMR modalities/applications. However, despite these notable technical advances, there is still limited evidence of their practical value and impact in real-world clinical settings. To help address this gap, we discuss a roadmap to translate AI CMR research into routine clinical practice, aiming to accelerate the adoption of these techniques and to ensure their promise can be realized in a fair, responsible, and equitable manner.

These considerations and recommendations emphasize the importance of big, high-quality, and representative datasets for training and testing AI models, recognizing the challenges in dataset size, diversity, and quality. The role of guidelines for reporting AI CMR research is also underscored to enhance study reproducibility and facilitate a better understanding of findings. Furthermore, the importance of generalizability, transparency, accountability, and explainability in the deployment of AI in CMR is highlighted. The article also touches upon industry considerations, pointing out several opportunities associated with the higher complexity and need for analysis of CMR, and acknowledging challenges related to data privacy, compatibility between different AI-based methods/approaches for different tasks and the importance of ensuring generalization and stable performance of products on a large scale. Last, it also provides insights into the US regulatory landscape, focusing on the US FDA's perspectives.

In conclusion, this article emphasizes the necessity of interdisciplinary collaboration between MR physicists, clinicians, AI scientists, and industry professionals to further advance AI CMR. This collaborative effort, coupled with rigorous regulatory and ethical considerations, is deemed essential to ensure the responsible and effective deployment of AI in routine clinical CMR workflows. The ongoing commitment of the SCMR community to prioritize these crucial aspects is therefore essential for the advancement of CMR through AI and for providing clinical evidence of their benefits.

## Author contributions

**Mariya Doneva:** Writing – review and editing, Methodology. **Jens Wetzl:** Writing – review and editing, Methodology. **Jana G. Delfino:** Writing – review and editing, Methodology. **Declan P. O’Regan:** Writing – review and editing, Methodology. **Claudia Prieto:** Writing – review and editing, Writing – original draft, Methodology, Conceptualization. **Frederick H. Epstein:** Writing – review and editing, Writing – original draft, Methodology, Conceptualization. **Qiang Zhang:** Writing – review and editing, Writing – original draft, Methodology. **Anastasia Fotaki:** Writing – review and editing, Writing – original draft, Methodology. **Sona Ghadimi:** Writing – review and editing, Writing – original draft, Methodology. **Yu Wang:** Writing – review and editing, Writing – original draft, Methodology.

## Declaration of competing interests

Qiang Zhang reports a relationship with British Heart Foundation that includes funding grants. Qiang Zhang has patent #Enhancement of Medical Images (WO/2021/044153) pending to Oxford University Innovation. Qiang Zhang has patent #Validation of quantitative magnetic resonance imaging protocols (WO2020234570A1) pending to Oxford University Innovation. Mariya Doneva is an employee of Philips GmbH Innovative Technologies, Hamburg, Germany. Mariya Doneva is an editorial board member of Magnetic Resonance Medicine and IEEE Transactions on Computational Imaging. Jens Wetzl is an employee and shareholder of Siemens Healthineers AG. DPO’R has consulted for Bayer and BMS on AI, holds a research grant from Bayer, and holds relevant patents. Claudia Prieto is an Associate Editor for Magnetic Resonance in Medicine and was not involved in the editorial review or the decision to publish this article. Frederick H. Epstein has research support from Siemens. Frederick H. Epstein is an editorial board member of JCMR and was not involved in the editorial review or the decision to publish this article. Frederick H. Epstein holds relevants patents. Sona Ghadimi holds relevant patents. Yu Wang holds relevant patents. The other authors declare that they have no known competing financial interests or personal relationships that could have appeared to influence the work reported in this paper.
